# Socially active neighborhoods: construct operationalization for aging in place, health promotion and psychometric testing

**DOI:** 10.1093/heapro/daac191

**Published:** 2023-02-16

**Authors:** Nestor Asiamah, Andrew Bateman, Peter Hjorth, Hafiz T A Khan, Emelia Danquah

**Affiliations:** Division of Interdisciplinary Research and Practice, School of Health and Social Care, University of Essex, Essex, Colchester CO4 3SQ, UK; Department of Gerontology and Geriatrics, Africa Centre for Epidemiology, Accra, Ghana; Division of Interdisciplinary Research and Practice, School of Health and Social Care, University of Essex, Essex, Colchester CO4 3SQ, UK; Institute of Regional Health Research, University of Southern Denmark, 5000 Odense, Denmark; College of Nursing, Midwifery and Healthcare, University of West London, Brentford TW8 9GB, UK; Department of Gerontology and Geriatrics, Africa Centre for Epidemiology, Accra, Ghana; Logistics and Supply Chain Management, School of Business, Koforidua Technical University, Koforidua, Ghana

**Keywords:** neighborhood walkability, neighborhood sociability, socially active neighborhoods, aging, aging in place, older adults

## Abstract

From the year 2003 when the first walkability scale was published to date, person-environment fit models and empirical research, some of which was published in *Health Promotion International*, have encapsulated healthy communities in ‘neighborhood walkability’. While there is no doubt that neighborhood walkability positively influences health-seeking behaviors and health, recent models suggest that their measurement and conceptualization have not emphasized the role played by psychosocial and personal factors in aging in place. Thus, the development of scales measuring human ecosystem factors has not recognized all critical factors suited for older adults. In this paper, we aim to draw on relevant literature to frame a more holistic construct, hereby referred to as Socially Active Neighborhoods (SAN), that would better support aging in place in older populations. Through a narrative review based on a systematic search of the literature, we define the scope of SAN and delineate some contextual implications for gerontology, health promotion and psychometric testing. SAN, unlike neighborhood walkability in its current measurement and conceptualization, incorporates critical theory-informed psychosocial factors (i.e. safety and disability friendliness of neighborhood infrastructure) that can encourage older adults with physiological and cognitive limitations to maintain physical and social activities as well as health in later life. The SAN is the result of our adaptation of key person-environment models, including the Context Dynamics in Aging (CODA) framework, that recognizes the role of context in healthy aging.

## INTRODUCTION

Over the last two decades, the term neighborhood walkability has been operationalized and measured in research as an embodiment of health-supporting environmental factors such as services, parks and esthetics (World Health Organization, 2018; Stokes, 2019; Colom *et al.*, 2020). Walkability concerns street connectivity, mixed land use (i.e. commercial and residential uses) and high residential density that facilitate easy access to services and other built environment attributes (Sallis *et al.*, 2010). Walkability encourages walking and other active behaviors (i.e. physical activity and social engagement) with its attributes such as parks, gardens and services (Asiaman *et al.*, 2021). Walkability plays an important role in aging in place as it provides built and psychosocial environment factors that encourage health-seeking behaviors over the life course (Asiamah *et al.*, 2021; Pani-Harreman *et al.*, 2021). Pani-Harreman *et al.* (2021) defined aging in place as maintaining physical functional capacity, independence and optimal health in one’s neighborhood or home while growing old.

Aging in place is an outcome of the interplay between three categories of factors, namely built environment attributes (i.e. streets, pavements, traffic flow) (Leyden, 2003; Lo, 2009; Sallis *et al.*, 2010; Asiamah *et al.*, 2022), psychosocial factors such as peace, trust and safety (Wahl and Gerstorf, 2018; Gan *et al.*, 2020; Asiamah, 2021) and personal factors including income, functional ability and age (Van Holle *et al.*, 2016; Asiamah, 2021). Recent commentaries (Wahl and Gerstorf, 2020; Asiamah, 2021) suggest that these three categories of factors play complementary roles in aging well in context. A built environment characterized by street connectivity, traffic and other architectural features that lack psychosocial factors such as peace and safety is unlikely to support aging in place, an idea that informed the development of the OpenX scale (Gan *et al.*, 2020), one of the few scales incorporating psychosocial factors. Even if such an environment offers all essential psychosocial factors, it may not support aging in place if its residents do not have the physical ability to utilize contextual attributes such as services (Wahl and Gerstorf, 2020; Asiamah, 2021). Hence, the framing and measurement of the best context to age should not undermine any of the above factors.

A recent scoping review by Almeida and colleagues (2021) suggests that neighborhood walkability, a construct assumed to measure places to age well (Lo, 2009; Battista and Manaugh, 2018), does not include the aforementioned psychosocial factors. In addition, some measures of the human ecosystem include psychosocial factors but in an inconsistent way (Asiamah *et al.*, 2020; Gan et al., 2020, 2022); each of these scales captured different psychosocial factors and most of them did not demonstrate or evidence the role of personal factors in age-friendly neighborhoods when being developed. So, a conceptual model that brings together the foregoing three factors in one place and demonstrates how personal variables interplay with built and psychosocial environment factors for aging in place was necessary.

Given the above concerns, we draw on relevant theories and models in environmental gerontology to: (i) propose a conceptual framework, hereby referred to as the *Socially Active Neighborhood (SAN)*; (ii) define and justify SAN as a more holistic construct of the ideal place for older adults and (iii) delineate implications for health promotion and psychometric testing. While some frameworks have been developed to demonstrate the role of psychosocial and personal factors in human ecosystems (Rogers *et al.*, 2013; Battista and Manaugh, 2018; Wahl and Gerstorf, 2018, 2020), our framework is unique for integrating key models developed to date, recognizing a gradual decline in physical functional ability in the aging process and providing a model that depicts the roles of psychosocial and personal factors in age-friendly neighborhoods for aging in place. A narrative review was employed because recent systematic reviews (Mazumdar *et al.*, 2018; Almeida *et al.*, 2021) provide necessary insights into measures of the above three categories of contextual factors, so there was no need for a complete systematic review to meet our aim. The following section is a summary of our systematic search methodology.

## SUMMARY OF THE NARRATIVE REVIEW PROCESS AND OUTCOMES

Our systematic search for articles followed the PRISMA (Preferred Reporting Items for Systematic Reviews and Meta-Analyses) guideline (see Supplementary material S1). The aim of the search was to identify and compile all peer-reviewed studies and publications focused on the measurement of neighborhoods in an ‘aging in place’ context. The inclusion and exclusion criteria, databases searched, search string and other relevant information are shown in the review workplan (see Supplementary material S2). As the PRISMA diagram shows, 32 articles were incorporated into this review. Information on items and domains included in all scales was extracted independently by two of the researchers (NA and ED) using a piloted data extraction Microsoft Excel sheet. These researchers then identified studies that examined the correlation between a relevant scale and personal factors.

Validated scales identified include the Neighbourhood Environment Walkability Scale (NEWS) (Saelens *et al.*, 2003), the OpenX tool (Gan *et al.*, 2020), the Physical Activity Neighbourhood Environment Scale (PANES) (Sallis *et al.*, 2010), the Availability of Built Environment Factors (ABEFs) scale (Asiamah *et al.*, 2020), the Rural Active Living Perceived Environmental Support Scale (RALPESS) by Umstattd *et al.* (2012), the Senior Walk Environment Assessment Tool (SWEAT) by Michael *et al.* (2009) and the more recent Walk/Wheelability scale (Gan *et al.*, 2022). The primary domains of the scales measuring neighborhoods in the context of aging in place were physical (built) factors (e.g. road signs, street connectivity, parks, pavements), psychosocial factors (e.g. safety, peace, community inclusiveness or cohesion, comfort) and behavioral factors (e.g. ease of walking). Safety was the most consistent psychosocial factor included in the tools. Only the ABEFs included safety and disability-friendliness of neighborhood infrastructure, though the SWEAT captured ‘the nature of buildings’ in the neighborhood. Moreover, only the ABEFs evidenced the potential correlation between personal factors and neighborhood attributes. Thus, the review confirms age-friendliness of neighborhoods as an outcome of the interplay between ecological, psychosocial and personal factors. We draw on this outcome to formulate SAN and its theoretical foundation in the next section.

## DEFINITION AND CONSTRUCTS OF THE SAN

### The theoretical basis of SAN

The role of the built environment in aging has been explained with bio-ecological and person-environment fit models. Environmental gerontology, a subfield of gerontology that concerns how healthy behaviors and health are chronologically shaped by environmental factors, was founded on the import of Lewin’s (1951) bio-ecological framework. This model premises that people and their behaviors (e.g. social and physical activities) are influenced by the environment. By this argument, the model sets the pace for other theoretical models that followed, a typical example being the person-environment fit model of Lawton and Simon (1968). With this framework, Lawton and Simon posited that residents and their neighborhoods are linked by some ‘demands’ characterized by the availability of built environment attributes such as services. Demands can be met by utilizing neighborhood resources through social engagement. This notion is consistent with Atchley’s (1971) continuity theory of aging which assumes that the maintenance of social interactions, physical activity and optimal health in context requires the ability to adapt previous life experiences. The ability to adapt previous experiences, which we refer to as *adaptation,* is associated with personal factors such as income, age and physical functional ability (Gan *et al.*, 2020; Asiamah, 2021). To illustrate, the aging person needs money to utilize services (e.g. healthcare, security services) in the neighborhood. They also need physical functional ability to walk around and socialize with others in the community. So, Lawton and Simon’s model and the continuity theory of aging both recognize physical functional ability and the ability to adapt past experiences as personal requirements for aging well in context.

Though very influential in environmental gerontology (Wahl and Gerstorf, 2018), Lawton and Simon’s (1968) model has been criticized for focusing on a disengaged state of older adults which undermines personal and environmental attributes that make healthy aging possible. This criticism spurred Lawton (1989) to develop his docility-proactivity model that premises that the willful utilization of contextual resources enables the individual to maintain physical functional capacity and health in the aging process. This reasoning has been supported by the life-space concept developed by Cantor (1975) based on the results of a survey carried out in New York. This concept argues based on empirical evidence that healthy behaviors such as walking are encouraged by neighborhood services and related factors. Apart from the life-space concept, the ecosystem model constructed by Bronfenbrenner (1979) also links the individual’s behavior (e.g. physical and social activities) to ecological and psychosocial factors. For example, the individual’s health status and the behaviors underlying it are influenced by ecosystem factors such as the family and community culture. The family and culture of the community directly determine the child’s behaviors (including healthy behaviors) and indirectly influence these behaviors by providing social support and other psychosocial factors (e.g. peace, trust) that encourage social inclusion in the aging process. A child who grows up with active parents in a sociable neighborhood characterized by psychosocial factors would value physical activity and, therefore, maintain neighborhood-level social and physical activities over the life course.

The CODA, an integrative model incorporating the above person-environment fit models, builds on the foregoing import of Bronfenbrenner (1979). Noteworthy is this model’s recognition of the central role of psychosocial factors (e.g. social support, safety, trust) in the creation of a sense of community. It contends that healthy aging occurs in contexts where built factors (e.g. street connectivity, high residential density, mixed land use), psychosocial factors and social capital enable individuals to maintain a lifelong trajectory of active behaviors, which results in the maintenance of optimal health into late life. Putting together the imports of the above theories, we can say that theories in gerontology recognize three categories of factors as the domains of the ideal place to age well. The first category hereby referred to as walkability, is recognized by all the above person-environment fit models as community design attributes (e.g. streets, parks, pavements, traffic lights) that are the core of the neighborhood walkability construct and its scales (e.g. NEWS and PANES). As revealed early on, this category encourages walking and other physical activities but would not support social inclusion in neighborhoods where violence, crime and other forms of social unrest are rife. An epidemic may also render walkable neighborhoods unattractive to residents, especially older adults with vulnerabilities as well as physical and cognitive limitations. Recent studies have revealed that violence and infectious diseases (e.g. Coronavirus disease 2019) cause social isolation (Tung *et al.*, 2019; Hwang *et al.*, 2020). So, the second category comprising psychosocial factors (i.e. trust, peace, social cohesion, the absence of an epidemic) is crucial for lifelong healthy behaviors as it is expected to encourage social engagement. This second component, sociability, is recognized by all the above person-environment fit models but is more elaborate in the CODA (Wahl and Gerstorf, 2018) and Bronfenbrenner’s (1979) model. By our observation, the CODA and some scales (e.g. OpenX, RALPESS, ABEFs), better emphasize the role of psychosocial factors in sociability as well as the impact of sociability on healthy aging. The third factor comprises personal factors (e.g. income, age, functional capacity) that define the individual’s ability to utilize neighborhood resources and adapt previous experiences. As discussed in the next section, therefore, person-environment fit theories recognize SAN as a more holistic proxy of the place to age well.

### Definition and framing of SAN

Drawing on our theoretical framework, especially the CODA, it is irrefutable that neighborhood walkability factors are relevant to healthy aging in place because essential services and related built environment attributes facilitate active behaviors among residents. The availability of these contextual factors encourages social and physical activities within groups and segments of the population, which is necessary for the maintenance of social capital (e.g. social support) for optimal health in the aging process (Faquinello and Marcon, 2011; Asiamah *et al.*, 2020). When services fuel active close-knit relationships as described above, individuals can maintain a spectrum of social and economic resources (e.g. physically active social ties, proximal ties, social ties that offer financial support) that benefit healthy aging in context. Other physical aspects of neighborhood design such as streets, traffic lights and parks boost a sense of community and impel people to visit places and participate in group activities through active forms of transportation such as walking, bicycling and skating. Suffice it to say that a community with a high residential density characterizes architectural design attributes (e.g. pavements, services, parks) and social resources (e.g. social support) that are in proximity. There is a consensus among researchers (Rivera *et al.*, 2010; De Grande, 2014; Levasseur *et al.*, 2015) that the proximity of contextual and social resources encourages and eases social engagement through active forms of transportation. In this vein, the proximity of resources holds much meaning in the context of older people who may be unable to reach far destinations through active transportation. Deductively, community design attributes that are the core of neighborhood walkability measures are fundamental to SAN, here referred to as neighborhood sociability.

In older populations, the sociability of the neighborhood is ultimately a combination of the foregoing physical design features and psychosocial factors. This reasoning is premised on the fact that physical walkability factors can only encourage older adults with physiological and cognitive limitations to socialize if the neighborhood is safe and contains the support needed (Faquinello and Marcon, 2011; Gan et al., 2020, 2022; Asiamah, 2021). Older adults who recognize their physical limitations and low ability to maneuver violent activities are unlikely to commute to settings prone to crime, violence, ageist stereotype and epidemics. Arguably, neighborhoods that lack psychosocial factors are volatile and, therefore, cannot support social inclusion over the life course in vulnerable segments of the population. We, therefore, reason that the worth of a neighborhood from the perspective of healthy aging largely draws on sociability, albeit walkability is a necessary complement to sociability. If so, the ideal place to age well is a sociable neighborhood comprising walkability and sociability attributes, which are defined and operationalized in Table 1.

**Table 1.** Operational definitions of potential domains of SAN^a^

SN	Domain	Definition (reference)	Key attributes/examples
Walkability			
1	High residential density	A compact neighborhood characterized by 12 to 25 dwelling units per gross acre of land (Mitrany, 2005)	The closeness of commercial and residential homes and businesses; proximity to services; proximity to social networks
2	Street connectivity	The interconnectedness of (spacious) streets and roads that connect to homes, shops, parks and neighborhood social centres (Molaei *et al.*, 2021)	T-, Y- and L-junctions, spacious streets (with pavements), cross-roads
3	Mixed land use	A balanced distribution of residential homes and commercial establishments in the neighborhood (Sallis *et al.*, 2010)	Residential homes, businesses to serve residents
Sociability^b^			
1	SDCI	Safety in the neighborhood and disability-friendliness of commercial infrastructure (Asiamah *et al.*, 2020)	Peace, safety within public space, disability-friendly commercial and residential buildings
2	Trust	A person’s strong belief in the reliability, truth or ability of someone (Baum and Ziersch, 2003)	Social engagement with others (including new friends); partnerships and collaborations
3	Social cohesion	The willingness of residents to cooperate to live in good health, survive and prosper (Martínez-Martínez *et al.*, 2018)	Keep-fit clubs, religious groups, credit unions
4	Social support	The physical, economic and emotional comfort given to people by their family, friends, co-workers and others (Baum and Ziersch, 2003)	Health information, assistance to access healthcare
5	Reciprocity	The practice of neighbors exchanging things of value with others for mutual benefit (Cagney and Wen, 2008).	Social support; volunteering

**Notes**: Like items in existing scales, the ‘key attributes/examples’ could be measured on a Likert-type scale that assesses availability, accessibility, relevance or quality of the neighborhood attributes. Most previous scales including the NEWS, PANES and ABEFs measure perceptions regarding the *availability* of the attributes with a Likert scale (i.e. not available, sometimes available, always available), which can be adopted for new measures. SAN, socially active neighborhoods; SN, serial number; SDCI, safety and disability-friendliness of commercial infrastructure.

^a^SAN are neighborhoods characterized by walkability and sociability attributes and, therefore, encourage and support adaptive behavior in the aging process.

^b^Sociability is the degree to which a neighborhood provides social support attributable to trust, cohesion and reciprocity for adaptive behavior in the aging process.

As our theoretical framework suggests, the primary domains of SAN are sociability and walkability. Social support, trust and safety are among the dimensions of sociability that create and sustain community bonds. As already captured in traditional walkability scales (Saelens *et al.*, 2003; Sallis *et al.*, 2010), factors such as street connectivity, mixed land use and high residential density are primary domains of walkability but are ideally secondary domains of SAN. We treat ‘safety and disability friendliness of commercial infrastructure’ as an indicator of safety and sanitation found in traditional walkability scales and recognize it as a psychosocial factor for two reasons. Firstly, the safety and disability friendliness of commercial infrastructure would inform decisions made by people, especially older adults, to socialize or make use of essential services and infrastructure with others. Secondly, the safety and disability-friendliness of commercial infrastructure (e.g. towers) are indicators of neighborhood safety. Due to the increasing rate of industrialization around the world (Parvaneh *et al.*, 2016; Yin *et al.*, 2020), we expect the safety and disability friendliness of commercial infrastructure to strongly underpin neighborhood safety and usability.

Recently, Asiamah (2022) averred how unsuitable neighborhoods would be for older adults when industrialization peaks, especially in developing countries. At the height of industrialization, residential neighborhoods would be transformed into official towns characterized by tall and complex commercial buildings. In a neighborhood with a high composition of skyscrapers, older adults may be unable to use community services and participate in social activities if lifts and other mobility support systems are not provided (Asiamah *et al.*, 2020; Asiamah, 2022). Consequently, older adults may be compelled to seek and remain in isolation. Deductively, the construct for aging well in place for older adults must include items or a factor measuring disability friendliness of neighborhood infrastructure.

**Figure 1.**
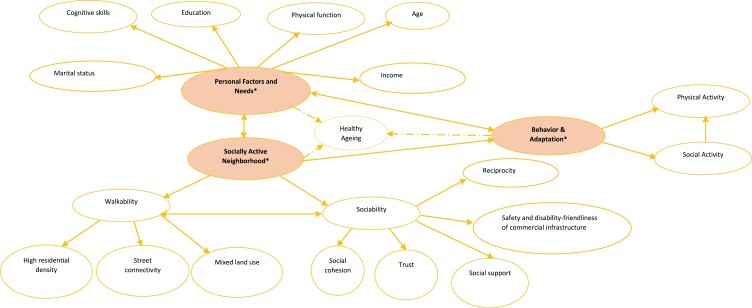
A conceptual framework of the relationship between a socially active neighborhood and healthy aging. *Ellipses with text (in bold) contain the primary variables; double-headed arrows represent correlations between the variables and broken arrows represent the influence of the primary variables on healthy aging.

Figure 1 is a framework of the three categories of factors that constitute SAN; this framework shows the relationships between these factors and healthy aging. As the figure indicates, personal factors are associated with adaptive behavior (i.e. the individual’s adaptive behavior such as social and physical activities), walkability and sociability. As the continuity theory of aging and CODA suggest, personal factors determine adaptive behavior through which walkability and sociability factors (e.g. disability friendliness of community infrastructure) are utilized. The relationships suggest that the ability of individuals to adapt previous experiences to use neighborhood resources including commercial infrastructure depends on personal factors such as age and physical function. So, the ideal place to age does not function in isolation from personal and psychosocial factors. That said, it is important to demonstrate the significance of SAN in the context of healthy aging and health promotion.

## THE SIGNIFICANCE OF SAN IN THE CONTEXT OF AGING IN PLACE

To reiterate, the continuity theory of aging premises that healthy aging occurs when the individual adapts past experiences to cope with and delay cognitive and physiological declines in the aging process. This theory, CODA, and the social capital theory of health (Cagney and Wen, 2008) further emphasize that adaptive behavior over the life course depends on social capital, environmental factors and individual attributes. We operationally define adaptive behavior as health-supporting behaviors (i.e. physical activity, social activity, healthy eating) maintained in the aging process against progressive declines in health and physical functional ability. As Figure 1 illustrates, adaptive behavior into late life is better supported in SAN, compared to walkable neighborhoods, the reason being that sociability provides social support and other psychosocial factors (e.g. disability friendliness of neighborhood infrastructure, safety, trust) that make adaptation possible or easier.

In Figure 1, the double-headed arrow between walkability and sociability connotes mutual support between the two domains. Thus, sociability is incomplete without walkability and vice versa. To substantiate this point, we would want to consider what a neighborhood with walkability but without sociability can offer aging people. The discussion so far suggests that such a neighborhood encourages social isolation and is less likely to support adaptive behavior, which is critical for aging well. Sociability alone does not offer the best support for healthy aging in place either, since walkability factors serve as physical resources in the community that impel people to engage in social participation and other adaptive behaviors. Thus, a community rich in trust, social cohesion and social support but void of walkable factors is unlikely to benefit healthy aging in place. No doubt putting sociability and walkability together gives rise to an ideal context for aging. This reasoning is consistent with studies that have demonstrated the positive effects of walkability and sociability factors on health indicators. Specifically, several studies (Chandrabose *et al.*, 2018; Kärmeniemi *et al.*, 2018; Tcymbal *et al.*, 2020) have evidenced positive effects of walkability factors (e.g. street connectivity, services) on long-term PA and health indicators. Empirical studies (Rothon *et al.*, 2011; Wang and Eccles, 2012; Asiamah *et al.*, 2020; Zimmer and McDonough, 2021) have shown that sociability factors such as social support as well as disability friendliness of neighborhood infrastructure have a positive effect on social and physical activities. In Figure 1, adaptive behavior, like SAN and personal factors, would directly influence healthy aging by supporting optimal health. SAN further influences healthy aging indirectly through adaptive behavior, which reflects the added value of SAN in aging in context. To appreciate the roles of SAN and adaptive behavior in the framework, ‘healthy aging’ could be replaced with ‘optimal health maintained over the life course’. These thoughts have implications for gerontology, health promotion and psychometric testing, which we discuss in the following section.

## IMPLICATIONS FOR HEALTH PROMOTION AND PSYCHOMETRIC TESTING

Our proposition of SAN as the ideal place to age well in older populations is a potential milestone in contextual gerontology as it identifies additional dimensions of an age-friendly neighborhood. As elucidated in the following paragraphs, an understanding of SAN is, thus, expected to inform new considerations and pathways in gerontology, health promotion and psychometric testing.

The idea that the built environment plays a crucial role in healthy aging cannot be disputed in the light of empirical and anecdotal evidence provided to date, but our proposition of SAN unfolds limitations fraught with how healthy neighborhoods have been framed and measured. A noteworthy limitation, which is addressed by our current operationalization of SAN, is the absence of key psychosocial factors in current measures of age-friendly contexts. The first step in advancing this effort of ours is taking steps to formalize SAN as an alternative to neighborhood walkability in a context where physiological limitations among residents may limit social and physical activities in the absence of relevant psychosocial factors such as the safety and disability friendliness of neighborhood infrastructure. Though SAN may not represent a full scope of the ultimate place for aging in place in every context, it might facilitate a better understanding of age-friendly neighborhoods and inform a productive debate toward generating ideas regarding places most suited for aging. In any case, future gerontological research adopting the most appropriate designs (e.g. cluster-randomized controlled trials) ought to investigate the relative effects of SAN and neighborhood walkability on long-term adaptive behavior and healthy aging. As plausible as our thoughts may be, their incorporation into practice as discussed later in this section should be based on empirical evidence showing that SAN, compared to neighborhood walkability, is more effective at maintaining optimal health in the aging process.

If future gerontological research confirms a positive longitudinal effect of SAN on adaptive behavior (e.g. physical and social activities) and demonstrates that SAN is more suited for aging in place than neighborhood walkability, then a basis is set for adopting SAN as the ideal place for aging in place over neighborhood walkability. This foundation would illustrate the worth of stakeholders’ investment in upgrading walkable neighborhoods into SAN, preserving sociable neighborhood factors for future generations and enhancing citizens’ knowledge about how to utilize and optimize SAN at the individual and community levels. Stakeholders (e.g. governments, civil society) should not only provide essential services and equip neighborhoods with recommended architectural and esthetic features but should also improve the disability friendliness of community infrastructure, safety, trust and social cohesion in neighborhoods. Neighborhoods that are already walkable but lack psychosocial factors can be upgraded into SAN by institutionalizing policies against community violence, crime, racial and ethnic stereotype, abuse of vulnerable groups (e.g. older adults, children) and inequality in employment, healthcare, as well as social welfare. The creation of SAN would include maximizing neighborhood safety by preventing the outbreak of epidemics or, at least, designing communities to support social activities during the spread of an epidemic. Enhancing neighborhood sanitation can prevent the outbreak of infectious disease and can be a panacea to residents’ fear of a pandemic. Within our thinking, health promotion is about balancing sociability and walkability in neighborhoods to optimize the individual’s ability to maintain healthy behaviors and health over the life course.

Since the neighborhood where the individual lives and ages is influenced by personal factors (e.g. income, gender, employment), this review implies that future validation of scales measuring age-friendly contexts should incorporate personal factors. An assessment of predictive validity in the form of a correlation between the personal factors and the scale as done in developing the ABEFs (Asiamah *et al.*, 2020) is a potential way to demonstrate the role of personal factors in SAN or related measures. Not demonstrating the role of personal factors in these measures can constrain health promotion efforts. The most effective health promotion programmes are person-centred (Ng *et al.*, 2015) and, therefore, draw on information about how personal variables relate to the issue being addressed. Secondly, inequalities in walkability and other measures of context stem from individual factors such as employment and income (Su *et al.*, 2017; Asiamah *et al.*, 2020), so recognizing the role of personal factors as suggested above could enable stakeholders to better understand and address these inequalities through health promotion or public health interventions. For instance, if living in more age-friendly neighborhoods is affected by income, health promotion programmes may aim to reduce income inequalities among older adults.

## LIMITATIONS WITHIN OUR THINKING

This paper does not empirically evidence the effects of SAN on physical activity, social activity and health; it only draws on theories and previous empirical evidence to propose SAN as the ideal context for aging in place. We also acknowledge that current scales measuring neighborhood walkability do not exclude all relevant psychosocial factors. These scales include items on safety such as sanitation and the absence of crime. The proposed structure and dimensionality of SAN are not empirically supported, which means that only the application of the right psychometric testing protocol can determine the composition of SAN and its proposed dimensions. Whether or not the few psychosocial factors captured in current walkability scales would better fit in sociability can only be determined with a recommended scale development protocol. We admit that a scale measuring SAN may be too long, but it is possible for psychometricians and gerontologists to develop a short version. Finally, this paper does not establish that sociability, compared to walkability, is more effective at supporting healthy aging; only a prospective design such as a cluster randomized controlled trial can evidence the relative effects of sociability and walkability on healthy aging.

## CONCLUSIONS

Neighborhoods play a role in healthy aging by providing access to both psychosocial and built environment factors that facilitate health-seeking behaviors and optimal health. Noteworthy is the idea that psychosocial factors are part of neighborhoods and are recognized by theories such as the CODA to complement built environment factors to enable people to age well in their communities. Without the psychosocial factors, walkable neighborhoods may be rendered uninhabitable by crime, racial and ethnic stereotypes, violence, epidemics and abuse of vulnerable groups. While walkable neighborhoods may be beneficial to younger people with the fortitude to navigate unsafe neighborhoods, they cannot support lifelong social participation and the maintenance of optimal health in older adults if they are prone to anti-social situations. As such, neighborhood walkability and its scales, which exclude most of the said psychosocial and personal factors, may not holistically encapsulate the best neighborhoods for aging in context. Disability friendliness of neighborhood infrastructure, social cohesion and social support are particularly implicit in sociability, which makes SAN a potentially better place to age well.

## Supplementary Material

daac191_suppl_Supplementary_Material

## References

[CIT0001] Almeida, D. P., Alberto, K. C. and Mendes, L. L. (2021) Neighborhood environment walkability scale: a scoping review. Journal of Transport and Health, 23, 1–12. doi:10.1016/j.jth.2021.101261.

[CIT0002] Asiamah, N. (2021). Socially active neighborhoods for older adults: a consolidation of evidence from psychometric tests and cross-sectional studies. *Published Doctoral Dissertation*. University of Portsmouth, School of Health and Care Professions, United Kingdom. https://researchportal.port.ac.uk/en/studentTheses/socially-active-neighbourhoods-for-older-adults

[CIT0003] Asiamah, N. (2022) Walkable urban neighborhoods: the adverse effects of industrialization and climate change in developing countries. In Chatterjee, U., Biswas, A., Mukherjee, J. and Mahata, D. (eds), Sustainable Urbanism in Developing Countries. CRC Press, United Kingdom, pp. 1–16.

[CIT0004] Asiamah, N., Kyriakos, K., Eduafo, R. and Borkey, R. (2020) The influence of community-level built environment factors on active social network size in older adults: social activity as a moderator. International Journal of Community Health Education, 41, 77–87. doi:10.1177/0272684X20915379. 32741318

[CIT0005] Asiamah, N., Lowry, R., Khan, H. T. A. and Awuviry-Newton, K. (2022) Associations between social support provided and walkability among older adults: health self-consciousness as a moderator. Archives of Gerontology and Geriatrics, 101, 104691. doi:10.1016/j.archger.2022.104691. 35339805

[CIT0006] Asiamah, N., Petersen, C., Kouveliotis, K. and Eduafo, R. (2021) The built environment and socio-demographic correlates of partial and absolute sedentary behaviours in community-dwelling older adults in Accra, Ghana. Journal of Cross-Cultural Gerontology, 36, 21–42. doi:10.1007/s10823-020-09417-5. 33141375

[CIT0007] Asiaman, N., Conduah, A. K. and Eduafo, R. (2021) Social network moderators of the association between Ghanaian older adults’ neighborhood walkability and social activity. Health Promotion International, 36, 1357–1367. doi:10.1093/heapro/daaa156. 33517412

[CIT0008] Atchley, R. C. (1971) Retirement and leisure participation: continuity or crisis? The Gerontologist, 11, 13–17. doi:10.1093/geront/11.1_part_1.13. 5579223

[CIT0009] Battista, G. A. and Manaugh, K. (2018) Stores and mores: toward socializing walkability. Journal of Transport Geography, 67, 53–60. doi:10.1016/j.jtrangeo.2018.01.004.

[CIT0010] Baum, F. E. and Ziersch, A. M. (2003) Social capital. Journal of Epidemiology and Community Health, 57, 320–323. doi:10.1136/jech.57.5.320. 12700212 PMC1732452

[CIT0011] Bronfenbrenner, U. (1979). The Ecology of Human Development: Experiments by Nature and Design. Harvard University Press, Cambridge, MA.

[CIT0012] Cagney, K. A. and Wen, M. (2008). Social capital and aging-related outcomes. In Kawachi, I., Subramanian, S. and Kim, D. (eds), Social Capital and Health. Springer, New York, NY, pp. 239–259. doi:10.1007/978-0-387-71311-3_11

[CIT0013] Cantor, M. H. (1975) Life space and the social support system of the inner city elderly of New York. The Gerontologist, 15, 23–27. doi:10.1093/geront/15.1_part_1.23. 1110000

[CIT0014] Chandrabose, M., Rachele, J. N., Gunn, L., Kavanagh, A., Owen, N., Turrell, G. et al. (2018) Built environment and cardio-metabolic health: systematic review and meta-analysis of longitudinal studies. Obesity Reviews, 20, 41–54. doi:10.1111/obr.12759. 30253075

[CIT0015] Colom, A., Mavoa, S., Ruiz, M., Wärnberg, J., Muncunill, J., Konieczna, J. et al. (2020) Neighborhood walkability and physical activity: the moderating role of a physical activity intervention in overweight and obese older adults with metabolic syndrome. Age and Aging, 50, 963–968. doi:10.1093/aging/afaa246. PMC8248320 33219673

[CIT0016] De Grande, P. (2014) Neighbors and neighborhood. Effects of proximity, educational and economic status on personal networks in Argentina. Delaware Review of Latin American Studies, 15, 1–27.

[CIT0017] Faquinello, P. and Marcon, S. S. (2011) Friends and neighbors: an active social network for adult and elderly hypertensive individuals. Revista da Escola de Enfermagem da USP, 45, 1345–1352. doi:10.1590/s0080-62342011000600010. 22241191

[CIT0018] Gan, D. R. Y., Fung, J. C. and Cho, I. S. (2020) Neighborhood experiences of people over age 50: factor structure and validity of a scale. The Gerontologist, 60, E559–E571. doi:10.1093/geront/gnz111. 31504478

[CIT0019] Gan, D. R. Y., Mahmood, A., Routhier, F. and Mortenson, W. B. (2022) Walk/wheelability: an inclusive instrument pair for participatory age-friendly research and practice. The Gerontologist, 62, E39–E47. doi:10.1093/geront/gnab079. 34164673

[CIT0020] Hwang, T. -J., Rabheru, K., Peisah, C., Reichman, W. and Ikeda, M. (2020) Loneliness and social isolation during the COVID-19 pandemic. International Psychogeriatrics, 32, 1217–1220. doi:10.1017/s1041610220000988. PMC7306546 32450943

[CIT0021] Kärmeniemi, M., Lankila, T., Ikäheimo, T., Koivumaa-Honkanen, H. and Korpelainen, R. (2018) The built environment as a determinant of physical activity: a systematic review of longitudinal studies and natural experiments. Annals of Behavioral Medicine, 52, 239–251. doi:10.1093/abm/kax043. 29538664

[CIT0022] Lawton, M. P. (1989) Environmental proactivity in older people. In Bengtson, V. L. and Schaie, K. W. (eds), The Course of Later Life. Springer, New York, NY, pp. 15–23.

[CIT0023] Lawton, M. P. and Simon, B. (1968) The ecology of social relationships in housing for the elderly. The Gerontologist, 8, 108–115. doi:10.1093/geront/8.2.108. 5657480

[CIT0024] Levasseur, M., Généreux, M., Bruneau, J. -F., Vanasse, A., Chabot, E., Beaulac, C. et al. (2015) Importance of proximity to resources, social support, transportation and neighborhood security for mobility and social participation in older adults: results from a scoping study. BMC Public Health, 15, 1–19. doi:10.1186/s12889-015-1824-0. 26002342 PMC4460861

[CIT0025] Lewin, K. (1951) Field Theory in Social Science. Harper and Brothers, New York, NY.

[CIT0026] Leyden, K. M. (2003) Social capital and the built environment: the importance of walkable neighborhoods. American Journal of Public Health, 93, 1546–1551. doi:10.2105/AJPH.93.9.1546. 12948978 PMC1448008

[CIT0027] Lo, R. H. (2009) Walkability: what is it? Journal of Urbanism, 2, 145–166. doi:10.1080/17549170903092867.

[CIT0028] Martínez-Martínez, O. A., Ramírez-López, A. and Rodríguez-Brito, A. (2018) Validation of a multidimensional social cohesion scale. Sociological Methods and Research, 49, 1–31. doi:10.1177/0049124118769112.

[CIT0029] Mazumdar, S., Learnihan, V., Cochrane, T. and Davey, R. (2018) The built environment and social capital: a systematic review. Environment and Behavior, 50, 119–158. doi:10.1177/0013916516687343.

[CIT0030] Michael, Y. L., Keast, E. M., Chaudhury, H., Day, K., Mahmood, A. and Sarte, A. F. I. (2009) Revising the senior walking environmental assessment tool. Preventive Medicine, 48, 247–249. doi:10.1016/j.ypmed.2008.12.008. 19136025 PMC5003601

[CIT0031] Mitrany, M. (2005) High density neighborhoods: who enjoys them? GeoJournal, 64, 131–140. doi:10.1007/s10708-005-4099-7.

[CIT0032] Molaei, P., Tang, L. and Hardie, M. (2021) Measuring walkability with street connectivity and physical activity: a case study in Iran. World, 2, 49–61. doi:10.3390/world2010004.

[CIT0033] Ng, M. K., Yousuf, B., Bigelowa, P. L. and Van Eerd, D. (2015) Effectiveness of health promotion programmes for truck drivers: a systematic review. Health Education Journal, 74, 270–286. doi:10.1177/0017896914533953.

[CIT0034] Pani-Harreman, K. E., Bours, G. J. J. W., Zander, I., Kempen, G. I. J. M. and Van Duren, J. M. A. (2021) Definitions, key themes and aspects of ‘ageing in place’: a scoping review. Ageing and Society, 41, 2026–2059. doi:10.1017/S0144686X20000094.

[CIT0035] Parvaneh, M., Hajipour, K. and Hosseinpour, M. (2016) Assessing impact of industrialization on urban expansion in surrounding cities (Case Study: Assalouyeh, Iran). Journal of Applied Sciences, 16, 167–177. doi:10.3923/jas.2016.167.177.

[CIT0036] Rivera, M. T., Soderstrom, S. B. and Uzzi, B. (2010) Dynamics of dyads in social networks: assortative, relational, and proximity mechanisms. Annual Review of Sociology, 36, 91–115. doi:10.1146/annurev.soc.34.040507.134743.

[CIT0037] Rogers, S. H., Gardner, K. H. and Carlson, C. H. (2013) Social capital and walkability as social aspects of sustainability. Sustainability (Switzerland), 5, 3473–3483. doi:10.3390/su5083473.

[CIT0038] Rothon, C., Goodwin, L. and Stansfeld, S. (2011) Family social support, community ‘social capital’ and adolescents’ mental health and educational outcomes: a longitudinal study in England. Social Psychiatry and Psychiatric Epidemiology, 47, 697–709. doi:10.1007/s00127-011-0391-7. 21557090 PMC3328685

[CIT0039] Saelens, B. E., Sallis, J. F., Black, J. B. and Chen, D. (2003) Neighborhood-based differences in physical activity: an environment scale evaluation. American Journal of Public Health, 93, 1552–1558. doi:10.2105/ajph.93.9.1552. 12948979 PMC1448009

[CIT0040] Sallis, J. F., Kerr, J., Carlson, J. A., Norman, G. J., Saelens, B. E., Durant, N. et al. (2010) Evaluating a brief self-report measure of neighborhood environments for physical activity research and surveillance: Physical Activity Neighborhood Environment Scale (PANES). Journal of Physical Activity and Health, 7, 533–540. doi:10.1123/jpah.7.4.533. 20683096

[CIT0041] Stokes, J. E. (2019) Implications of perceived neighborhood quality, daily discrimination, and depression for social integration across mid- and later life: a case of person-environment fit? The Gerontologist, 59, 1–11. doi:10.1093/geront/gnz103. 31375829 PMC7228417

[CIT0042] Su, S., Pi, J., Xie, H., Cai, Z. and Weng, M. (2017) Community deprivation, walkability, and public health: highlighting the social inequalities in land use planning for health promotion. Land Use Policy, 67, 315–326. doi:10.1016/j.landusepol.2017.06.005.

[CIT0043] Tcymbal, A., Demetriou, Y., Kelso, A., Wolbring, L., Wunsch, K., Wäsche, H. et al. (2020) Effects of the built environment on physical activity: a systematic review of longitudinal studies taking sex/gender into account. Environmental Health and Preventive Medicine, 25, 1–25. doi:10.1186/s12199-020-00915-z. 33246405 PMC7697377

[CIT0044] Tung, E. L., Hawkley, L. C., Cagney, K. A. and Peek, M. E. (2019) Social isolation, loneliness, and violence exposure in urban adults. Health Affairs, 38, 1670–1678. doi:10.1377/hlthaff.2019.00563. 31589531 PMC7229645

[CIT0045] Umstattd, M. R., Baller, S. L., Hennessy, E., Hartley, D., Economos, C. D., Hyatt, R. R. et al. (2012) Development of the rural active living perceived environmental support scale (RALPESS). Journal of Physical Activity and Health, 9, 724–730. doi:10.1123/jpah.9.5.724. 21946157

[CIT0046] Van Holle, V., Van Cauwenberg, J., De Bourdeaudhuij, I., Deforche, B., Van de Weghe, N. and Van Dyck, D. (2016) Interactions between neighborhood social environment and walkability to explain Belgian older adults’ physical activity and sedentary time. International Journal of Environmental Research and Public Health, 13, 569. doi:10.3390/ijerph13060569. 27338426 PMC4924026

[CIT0047] Wahl, H. -W. and Gerstorf, D. (2018) A conceptual framework for studying context dynamics in aging (CODA). Developmental Review, 50, 155–176. doi:10.1016/j.dr.2018.09.003.

[CIT0048] Wahl, H. -W. and Gerstorf, D. (2020) Person–environment resources for ageing well: environmental docility and life space as conceptual pillars for future contextual gerontology. The Gerontologist, 60, 368–375. doi:10.1093/geront/gnaa006. 32240292

[CIT0049] Wang, M. T. and Eccles, J. S. (2012) Social support matters: longitudinal effects of social support on three dimensions of school engagement from middle to high school. Child Development, 83, 877–895. doi:10.1111/j.1467-8624.2012.01745.x. 22506836

[CIT0050] World Health Organization. (2018) *The Global Network for Age-friendly Cities and Communities: Looking Back Over the Last Decade, Looking Forward to the Next*. World Health Organization, Geneva, Switzerland. Retrieved July 26, 2021 from https://apps.who.int/iris/bitstream/handle/10665/278979/WHO-FWC-ALC-18.4-eng.pdf?sequence=1

[CIT0051] Yin, J., Zhao, X., Zhang, W. and Wang, P. (2020) Rural land use change driven by informal industrialization: evidence from Fengzhuang Village in China. Land, 9, 190–117, 10.3390/land9060190.

[CIT0052] Zimmer, C. and McDonough, M. H. (2021) Social support and physical activity in older adults: identifying predictors using data from the Canadian Longitudinal Study on Aging. Journal of Aging and Physical Activity, 30, 136–147. doi:10.1123/japa.2020-0393. 34348225

