# Supplementary material for: Socially active neighborhoods: construct operationalization for aging in place, health promotion and psychometric testing
Source: Health Promot Int. 2023 Feb 16;38(1):daac191. doi: 10.1093/heapro/daac191 (PMC9933838; doi:10.1093/heapro/daac191)
Supplement: daac191_suppl_Supplementary_Material [file daac191_suppl_supplementary_material.doc]

**Supplementary Material 1. Review Protocol**

| **1.BACKGROUND** | |
| --- | --- |
| Review Title | Validated measures of neighbourhoods supporting ageing in place |
| Start and End Date | August 1, 2020, to July 31, 2022 (2 searches were done between 2020 and 2021, and another one performed in 2022 for potentially new scales). The third search filtered for articles published between 2021 and July 31, 2022 to filter out articles previously used. |
| Research questions | (1) What are the domains of validated scales measuring neighbourhoods for ageing in place, and (2) has any study demonstrated the correlation between a scale and personal factors? |
| Condition or domain being study | Neighbourhood attributes for ageing in place |
| **2. SEARCH STRATEGY** | |
| **Eligibility Criteria (Based on PICO)** |  |
| *Population* | Older adults aged 50 years or higher or the general population including this group |
| *Intervention* | Psychometric testing |
| *Comparator* | Walkability |
| *Outcome* | Scales including built neighbourhood features, psychosocial and behavioural factors, and personal factors |
| *Language* | English |
| *Date restrictions* | No date restriction |
| *Exclusion Criteria* | Published in other languages apart from English, studies not  peer-reviewed, and studies not reporting traditional psychometric/scale validation procedures. |
| *Inclusion Criteria* | 1. Published in English 2. Studies focused on the development, validation, or translation of scales measuring neighbourhoods for ageing in place 3. Peer-reviewed |
| *Geographical scope* | Studies conducted anywhere in the world |
| **Databases** |  |
| *Essential* | Cochrane CENTRAL, PubMed, ProQuest |
| *As relevant to the subject:* | PsycInfo, CINAHL, SCOPUS |
| *Search String* | (((((Neighbourhood OR "built environment" OR community OR context OR place) AND ("Physical activity" OR exercise OR walking)) AND (Walkability OR "neighbourhood cohesion" OR "community cohesion" OR "age-friendly neighbourhood" OR "age-friendly community")) AND ("Social participation" OR "social activity" OR "social engagement")) AND ("Ageing in place" OR "ageing in context" OR "ageing in the community")) AND ("Measurement scale" OR "psychometric scale" OR questionnaire OR survey OR "psychometric tool") |

Note: PICO – Population, Intervention, Comparison, Outcome

Articles identified through database searches (n = 168 on PubMed; n = 201 on SCOPUS; n = 302 on ProQuest, and n = 62 on PsychInfo)

Full text articles retrieved for further review (n = 156)

Articles considered in the narrative review (n = 32)

Articles excluded based on abstract/title screening ((n = 134 on PubMed; n = 155 on SCOPUS; n = 238 on ProQuest, and n = 50 on PsychInfo)

Articles excluded based on the full review for relevance (n = 124)

Appendix 2. The PRISMA (search) diagram of included and excluded articles

Appendix 2. The PRISMA (search) diagram of included and excluded articles
